# The Effectiveness and Safety of Tai Chi on Knee Pain: A Systematic Review and Meta-Analysis

**DOI:** 10.3390/healthcare13131615

**Published:** 2025-07-06

**Authors:** Hyunggon Lee, Soo-Hyun Sung, Sangnam Lee

**Affiliations:** 1 Department of Qigong, College of Korean Medicine, Daegu Haany University, Gyeongsan 38609, Republic of Korea; gony1978@naver.com; 2 Department of Policy Development, National Institute for Korean Medicine Development, Seoul 04516, Republic of Korea

**Keywords:** Tai Ji, mind–body therapies, exercise movement techniques, randomized controlled trial, clinical evidence

## Abstract

**Background/Objectives:** Although Tai Chi has shown potential benefits for managing chronic pain, its clinical effectiveness specifically for knee pain remains inconclusive. **Methods:** We systematically searched ten electronic databases for randomized controlled trials (RCTs) investigating the effects of Tai Chi on knee pain. **Results:** This systematic review and meta-analysis included 11 RCTs involving 706 participants; among them, three studies (n = 169) were eligible for meta-analysis. A comprehensive search of ten electronic databases was conducted up to March 2025. The included RCTs were conducted in the United States (n = 5), China (n = 3), South Korea (n = 2), and Turkey (n = 1). Compared to health education, Tai Chi significantly improved knee pain, as measured using the Western Ontario and McMaster Universities Osteoarthritis Index (WOMAC) pain score (mean difference (MD) = −0.60; 95% CI: −6.52 to −3.28; *p* < 0.00001) and the Visual Analogue Scale (VAS) (MD = −1.44; 95% CI: −1.95 to −0.93; *p* < 0.00001). Tai Chi also significantly improved knee function compared to health education (WOMAC function score—MD = −13.49; 95% CI: −17.11 to −9.87; *p* < 0.00001). Four RCTs comparing Tai Chi with no intervention reported favorable effects on knee pain and function; however, a meta-analysis was not possible due to limited data. In contrast, two studies comparing Tai Chi with active controls, such as physical therapy and resistance training, found no significant differences in pain or functional outcomes. Two studies reported increased knee pain during initial Tai Chi sessions, but no adverse events occurred after postural corrections. **Conclusions:** While Tai Chi appears promising for knee pain management, further large-scale, high-quality RCTs with rigorous methodology are needed to establish definitive evidence.

## 1. Introduction

Tai Chi is a mind–body exercise that combines slow, controlled movements with focused mental discipline and is traditionally practiced to enhance overall health and well-being [1,2]. It is based on the philosophical principles of Taiji, which describe the interaction of opposing but complementary forces, regarded as the foundation of balance and harmony [1]. As a type of qigong, Tai Chi emphasizes the circulation of qi (vital energy) via coordinated movement, breath control, and mindfulness [1]. Tai Chi is typically performed in a semi-squatting posture with fluid, continuous motions. It does not require special equipment or facilities, making it accessible to people of all ages and fitness levels [2,3]. Practitioners are encouraged to focus on movement quality, which promotes relaxation, reduces stress, and enhances body awareness [1].

Accumulating evidence supports the therapeutic benefits of Tai Chi for various chronic conditions, such as musculoskeletal pain, cardiovascular disease, and mental health disorders [2,3]. Regular practice improves aerobic capacity, muscle strength, balance, flexibility, and quality of life, while also alleviating anxiety and depression [4,5,6,7,8,9,10,11]. These multidimensional effects highlight Tai Chi as a safe, low-impact, and holistic intervention, especially for individuals with chronic pain or functional limitations. Among these conditions, knee pain is one of the most common and burdensome complaints in adults.

Knee pain is one of the most common musculoskeletal complaints in adults, affecting approximately 25% of the population. Its prevalence has increased by nearly 65% over the past two decades [12,13,14]. It can result from various factors, including underlying conditions such as osteoarthritis, as well as physical activity, weight gain, and aging. The mechanisms underlying knee pain are multifactorial, involving both peripheral and central processes. At the tissue level, pain may arise from mechanical overload, injury, abnormal biomechanics, or degenerative changes, such as those associated with osteoarthritis [15,16]. Over time, about 86% of individuals with knee pain develop knee osteoarthritis, which is characterized by pain, stiffness, and swelling, ultimately resulting in functional impairment and limitations in daily activities [17].

Clinically, knee pain significantly impacts quality of life, physical function, and healthcare utilization. It is a leading cause of disability worldwide, restricting mobility and daily activities [13]. Moreover, pain and depression are closely linked through shared neurobiological pathways, and chronic pain is a significant risk factor for depression [18]. Therefore, the management of knee pain should not focus solely on symptom relief, but should also aim to improve daily functioning, social participation, interpersonal relationships, and mental health outcomes [19].

Conventional pain management strategies primarily include pharmacological and surgical interventions [20]. Commonly prescribed analgesics are acetaminophen, nonsteroidal anti-inflammatory drugs (NSAIDs), tramadol, and opioids. Although these medications are effective for pain relief, their benefits are often modest, and they are associated with a variety of adverse effects. Due to these limitations, there has been growing interest in complementary therapies as alternative or adjunctive treatments [21]. Of the various non-pharmacological interventions (e.g., yoga, resistance training, and aquatic therapy), Tai Chi stands out because it does not require specialized equipment or facilities, making it highly accessible [22]. It is especially suitable for older adults or those unable to tolerate high-intensity or weight-bearing exercise, providing a gentle yet effective option for pain management and functional improvement [23].

Kang et al. [24] systematically evaluated the effects of different Tai Chi parameters—such as session duration, frequency, and style—on outcomes in patients with chronic lower back pain. While this meta-analysis offered valuable insights into optimal practice parameters, its findings are limited to lower back pain and may not be generalizable to other musculoskeletal conditions, such as knee pain. Kong et al. [25] assessed the effectiveness of Tai Chi across several chronic pain conditions, including osteoarthritis, lower back pain, and fibromyalgia. Although this comprehensive meta-analysis demonstrated overall benefits in pain reduction and functional improvement, it did not provide condition- or joint-specific analyses, and few studies focused exclusively on knee pain.

To date, no systematic reviews have specifically evaluated the efficacy of Tai Chi for knee pain. Therefore, the aim of this review was to assess the effectiveness and safety of Tai Chi in reducing knee pain.

## 2. Materials and Methods

This systematic review was conducted following the Preferred Reporting Items for Systematic Reviews and Meta-Analyses (PRISMA) guidelines [26].

### 2.1. Protocol and Registration

The review protocol was registered with the International Prospective Register of Systematic Reviews (PROSPERO; registration number: CRD420251056546).

### 2.2. Data Sources and Searches

A comprehensive literature search was performed in the following electronic databases for studies published up to March 2025: PubMed, CINAHL Plus, EMBASE, Cochrane Central Register of Controlled Trials (CENTRAL), ScienceON, Korean Traditional Knowledge Portal, Korea Citation Index (KCI), Research Information Sharing Service (RISS), Oriental Medicine Advanced Searching Integrated System (OASIS), and the Korean Medical Database. No language restrictions were applied during study selection.

The search terms were as follows: (“Tai Ji” OR “Tai Chi” OR “Tai Chi Chuan” OR “Taiji” OR “Taijiquan” OR “T’ai Chi” OR “Tai Ji Quan”) AND (“knee pain”) AND (“clinical trial” OR “randomized controlled trial”). Detailed search strategies for each database are provided in Supplementary Table S2.

### 2.3. Inclusion/Exclusion

#### 2.3.1. Types of Studies

Randomized controlled trials (RCTs) evaluating the effectiveness and safety of Tai Chi for knee pain management were included. Studies were excluded if they were non-randomized, single-arm case reports without control groups, conference abstracts, review articles, surveys, or study protocols.

#### 2.3.2. Participants

Studies including participants with knee pain, regardless of underlying etiology, were eligible. No restrictions were applied regarding sex, age, ethnicity, or race.

#### 2.3.3. Types of Interventions

All forms of Tai Chi interventions were eligible for inclusion, with no restrictions on style, structure, duration, or frequency.

#### 2.3.4. Types of Comparisons

There were no limitations regarding the type of control intervention. Acceptable comparators included no-treatment controls, sham interventions, and active treatments (e.g., physical therapy and resistance training).

#### 2.3.5. Types of Outcome Measures

The primary outcomes were validated pain assessment tools, including the Visual Analogue Scale (VAS) and the Western Ontario and McMaster Universities Osteoarthritis Index (WOMAC). The WOMAC is widely used to assess pain, joint stiffness, and physical function in patients with osteoarthritis, making it suitable for evaluating interventions targeting knee pain. The VAS provides a simple and quantifiable measure of pain intensity. Secondary outcomes included functional assessments (e.g., range of motion (ROM)), muscle strength, joint stiffness, quality of life, proprioception, and adverse events.

### 2.4. Study Selection

Two authors (H.L. and S.-H.S.) independently screened titles and abstracts to identify studies meeting the inclusion criteria. Full-text articles of potentially eligible studies were then reviewed in detail. Discrepancies were resolved by discussion with a third author (S.L.).

### 2.5. Data Extraction

Two independent reviewers (H.L. and S.-H.S.) extracted data to minimize bias and enhance accuracy. The extracted information included study characteristics (first author, publication year, country, study design, sample size, participant demographics, and inclusion/exclusion criteria), detailed descriptions of interventions and comparators (type, frequency, duration, intensity, and structure of both Tai Chi and control programs), and outcome measures (primary and secondary endpoints, definitions, and assessment tools). The main findings such as effect sizes, confidence intervals, and statistical significance were systematically recorded, along with any reported adverse events or safety outcomes. For Tai Chi interventions, specific parameters (style, instructor credentials, session duration, total number of sessions, group versus individual formats, and participant adherence) were also extracted. When available, data on follow-up periods, dropout rates, and reasons for attrition were collected. Discrepancies between reviewers were resolved through discussion and consensus with a third reviewer (S.L.)

### 2.6. Assessment of Risk of Bias (ROB)

The methodological quality of the included RCTs was assessed using the Revised Cochrane Risk of Bias Tool (RoB 2.0) [27]. The following five domains were evaluated: (1) bias arising from the randomization process; (2) bias due to deviations from intended interventions; (3) bias due to missing outcome data; (4) bias in the measurement of outcomes; and (5) bias in the selection of reported results. Each domain and the overall risk of bias were rated as “low risk,” “some concerns,” or “high risk.” Two reviewers (H.L. and S.-H.S.) independently assessed the risk of bias. Prior to assessment, reviewers underwent calibration exercises to ensure consistency in applying the RoB 2.0 criteria. Disagreements were resolved by discussion and consensus; if consensus was not reached, a third reviewer was consulted.

### 2.7. Safety Assessment

All adverse events reported in the included studies were evaluated and graded according to the Common Terminology Criteria for Adverse Events (CTCAE) version 5.0 [28]. Adverse events were classified as follows: Grade 1 (mild)—asymptomatic or mild symptoms not requiring intervention; Grade 2 (moderate)—requiring minimal, local, or noninvasive intervention and potentially interfering with daily activities; Grade 3 (severe)—requiring hospitalization or the prolongation of hospitalization and significantly limiting self-care; Grade 4 (life-threatening)—requiring urgent intervention; and Grade 5 (death)—fatal events directly related to the adverse outcome. This standardized classification enabled a rigorous and objective assessment of the safety of Tai Chi interventions.

### 2.8. Data Analyses

Meta-analyses were performed using Review Manager (RevMan) version 5.4 (The Cochrane Collaboration, Nordic Cochrane Centre, Copenhagen, Denmark). For continuous outcomes, the results were reported as mean differences (MDs) with 95% confidence intervals (CIs). Statistical heterogeneity was assessed using the I^2^ statistic, with thresholds of >30% indicating moderate heterogeneity, >50% indicating substantial heterogeneity, and >75% indicating considerable heterogeneity. When meta-analysis was not feasible due to high heterogeneity or insufficient data, a narrative synthesis was conducted.

## 3. Results

### 3.1. Study Selection and Description

After duplicates were removed, 97 articles remained. Following title and abstract screening, 73 articles were excluded, leaving 24 full-text articles for eligibility assessment. Of these, 13 were excluded for the following reasons: study protocols (n = 2), case series (n = 1), non-randomized controlled trials (n = 4), and single-arm studies (n = 6). Consequently, 11 randomized controlled trials (RCTs) [29,30,31,32,33,34,35,36,37,38,39] met the inclusion criteria and were included in this systematic review.

**Figure 1:**
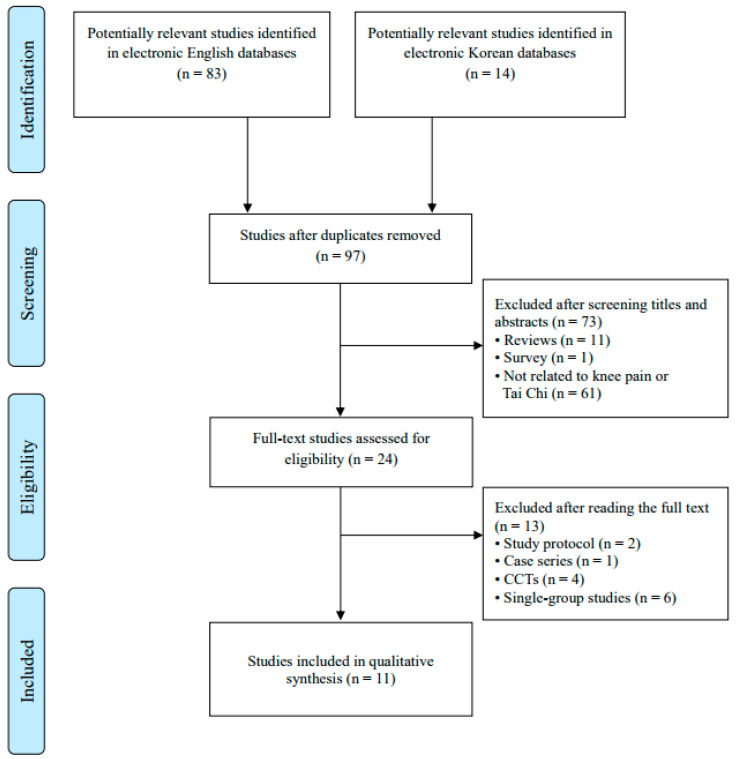
Flowchart of the study selection process. CCTs—controlled clinical trials.

The study selection process is shown in Figure 1. Among the included RCTs, five were conducted in the United States, three in China, two in South Korea, and one in Turkey. The detailed characteristics of the included studies are summarized in Table 1.

### 3.2. Participants

A total of 706 participants were enrolled in 11 RCTs, with 381 assigned to experimental groups and 335 assigned to control groups. Ten studies [29,30,31,32,33,34,35,36,38,39] included participants with knee osteoarthritis and related knee pain, whereas one study enrolled individuals with partial anterior cruciate ligament (ACL) injury and knee pain.

### 3.3. Intervention

Six trials [30,32,33] used the Yang style of Tai Chi, while two studies [29] used the Sun style. The remaining three studies did not specify the style of Tai Chi. Intervention durations ranged from 6 to 24 weeks (mean: 13.5 weeks), and the number of sessions ranged from 16 to 72 (mean: 38.7 sessions). Detailed descriptions of the Tai Chi programs are summarized in Table 2.

### 3.4. Control Interventions

The control interventions included health education [30,34,36,38,39], no intervention [29,31,33,37], stretching combined with health education [32], resistance training [33], and physical therapy [35]. One study [33] included two separate control groups. Detailed descriptions of the active control interventions [32,33,35] are presented in Supplementary Table S3.

### 3.5. Outcomes

#### 3.5.1. Tai Chi Versus Health Education

Five studies [30,34,36,38,39] compared Tai Chi with health education to assess its effects on knee pain, physical function, and stiffness. Four studies [30,34,36,38] reported that Tai Chi significantly reduced pain compared to health education, while one study [39] found no statistically significant difference between the two groups.

**Figure 2:**
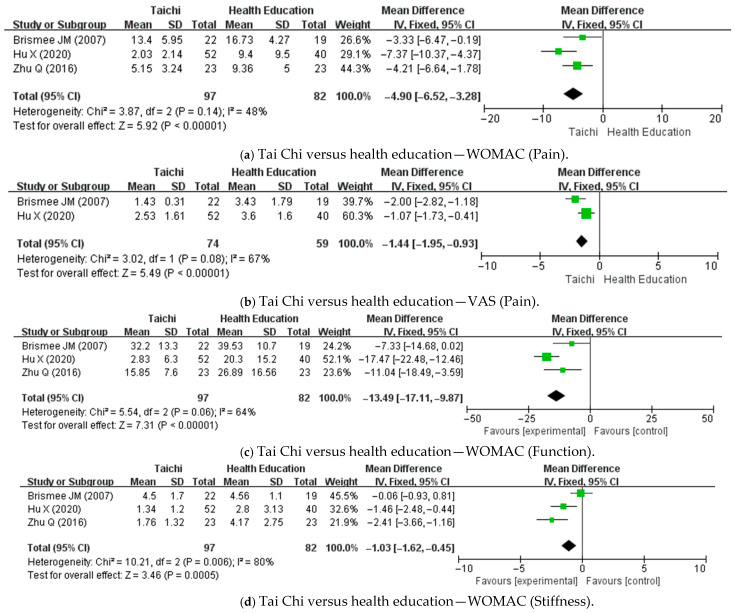
Meta-analysis of Tai Chi for knee pain, function, and stiffness compared with health education. CI: confidence interval; VAS: Visual Analogue Scale; WOMAC: Western Ontario and McMaster Universities Osteoarthritis Index [30,36,39].

Figure 2 presents forest plots summarizing the results of the meta-analysis comparing Tai Chi with health education for knee pain, physical function, and stiffness. In each panel, the x-axis represents the MD between the Tai Chi and control groups for each outcome. The vertical line at zero indicates the ‘line of no effect,’ representing no difference between the groups.

A meta-analysis of three RCTs [30,36,39] demonstrated a significant reduction in pain in the Tai Chi group compared with the control group, as measured using the WOMAC pain score (MD: −4.90; 95% CI: −6.52 to −3.28; *p* < 0.00001; n = 97; heterogeneity: I^2^ = 48%; Figure 2a). In two trials [30,39], Tai Chi also led to significantly greater improvements in pain severity, as measured using the VAS, compared to health education (MD: −1.44; 95% CI: −1.95 to −0.93; *p* < 0.00001; n = 74; heterogeneity: I^2^ = 67%; Figure 2b). Four studies [30,36,38,39] reported significant improvements in knee function in the Tai Chi groups relative to the controls, whereas one study [34] found no significant difference. A meta-analysis of three studies [30,36,39] showed a statistically significant improvement in function, as measured using the WOMAC function score (MD: −13.49; 95% CI: −17.11 to −9.87; *p* < 0.00001; n = 97; heterogeneity: I^2^ = 64%; Figure 2c). Similarly, a meta-analysis of the same three studies [30,36,39] revealed that Tai Chi significantly reduced knee stiffness compared to control groups (WOMAC stiffness score—MD: −1.03; 95% CI: −1.62 to −0.45; *p* = 0.0005; n = 97; heterogeneity: I^2^ = 80%; Figure 2d).

#### 3.5.2. Tai Chi Versus No Intervention

Four RCTs [29,31,33,37] assessed the effects of Tai Chi compared to no intervention on knee pain and functional outcomes. Among them, three studies [29,31,37] reported statistically significant reductions in knee pain in the Tai Chi group relative to controls. In contrast, one study [33] did not observe significant improvements in pain in either the intervention or control group.

Regarding functional outcomes, one RCT [29] demonstrated a significant improvement in physical function following Tai Chi intervention. However, the other three trials [31,33,37] did not find significant differences between the Tai Chi and control groups.

#### 3.5.3. Tai Chi Versus Stretching and Health Education

One RCT [32] reported a significantly greater pain reduction in the Tai Chi group compared to the group receiving stretching exercises combined with health education. Regarding functional outcomes, significant between-group differences were observed in the WOMAC function score and chair stand time. However, no statistically significant differences were reported for the walk test or balance assessments.

#### 3.5.4. Tai Chi Versus Physical Therapy

One RCT [35] compared Tai Chi with physical therapy and found no statistically significant differences in pain reduction or functional improvement between the two groups, indicating that both interventions were similarly effective.

#### 3.5.5. Tai Chi Versus Resistance Training

Wortley et al. [33] conducted an RCT evaluating the effects of Tai Chi versus resistance training on knee pain and function, as measured using the WOMAC score. Resistance training led to statistically significant improvements in both pain and function, whereas no significant improvements were observed in the Tai Chi group.

### 3.6. Safety of Tai Chi

Of the eleven included RCTs, six studies [30,32,34,35,36,38] reported data on adverse events (AEs). Four studies [34,35,36,39] reported no adverse events associated with Tai Chi. In two studies [30,32], participants experienced transient exacerbations of knee pain during the initial sessions. However, following postural correction, no further AEs were observed. These events were classified as Grade 1 according to the CTCAE.

### 3.7. Assessment for ROB

**Figure 3:**
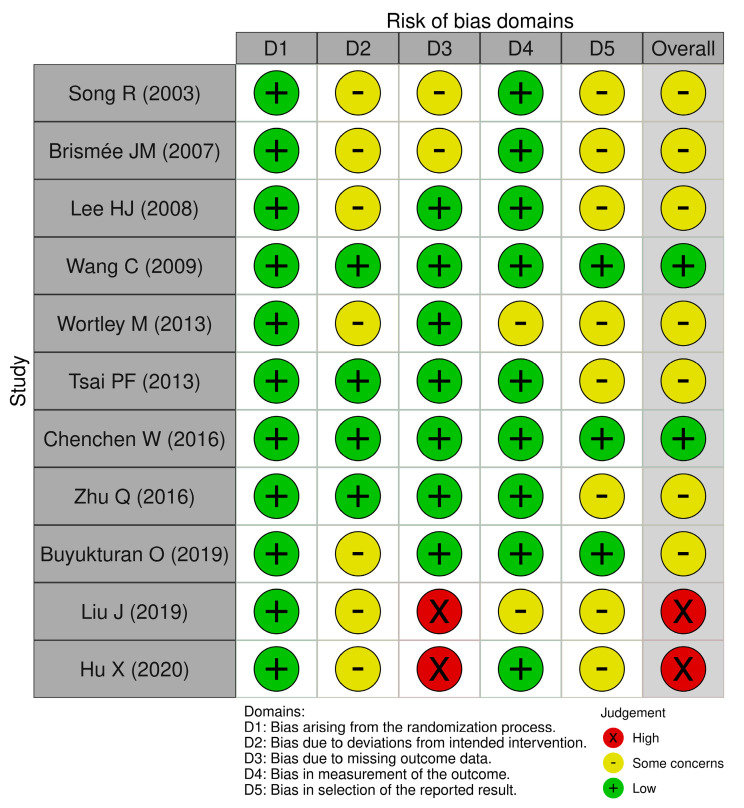
Risk of bias assessment. High: high risk of bias; L: low risk of bias [29,30,31,32,33,34,35,36,37,38,39].

Among the 11 included RCTs [29,30,31,32,33,34,35,36,37,38,39], the overall risk of bias (ROB) was rated as low in 2 studies (18.2%), as having some concerns in 7 studies (63.6%), and as high in 2 studies (18.2%) (Figure 3). Two studies [38,39] were judged to have a high risk of bias in the “missing outcome data” domain due to dropout rates exceeding 20% and the likelihood that the missing data were related to the true outcomes of the interventions.

## 4. Discussion

### 4.1. Main Findings

To address the study objective of evaluating the comparative effectiveness of Tai Chi, we systematically analyzed outcomes according to the type of control group (health education, no intervention, and active controls). Tai Chi consistently demonstrated significant improvements in knee pain and function compared to health education and no intervention, as evidenced by reductions in WOMAC and VAS scores. However, when compared with active control interventions—such as physical therapy and resistance training—the results differed. In these head-to-head trials, no statistically significant differences were observed between Tai Chi and active controls in terms of pain reduction or functional improvement, suggesting that Tai Chi is as effective as, but not superior to, established exercise-based therapies.

The pain-relieving effects of Tai Chi may be attributed to its combined influence on both peripheral and central pain pathways. Peripherally, the slow, controlled movements performed in semi-squatting postures enhance proprioception and muscular coordination, thereby reducing mechanical stress on the knee joint. Biomechanical analyses indicate that Tai Chi generates knee joint reaction forces approximately 1.2 times the body weight, which is substantially lower than those observed during walking (3–4 times the body weight), contributing to reduced joint loading and improved joint stability [40]. Centrally, Tai Chi integrates mindful movement with breath regulation, which may modulate descending inhibitory pain pathways and attenuate central sensitization. Significant improvements in depression scores and self-efficacy further suggest neuroplastic adaptations within pain-related neural circuits, supporting the role of Tai Chi in centrally mediated pain modulation [41].

When compared to active interventions such as resistance training or physical therapy [33,35], Tai Chi did not consistently demonstrate superior effects on pain or function. This similarity in outcomes may be attributed to shared components among these interventions, including muscle strengthening, joint mobilization, and balance training, all of which are fundamental for improving knee pain and function. Therefore, the distinct advantage of Tai Chi may lie in its accessibility, safety, and holistic approach, rather than in unique physiological effects beyond those provided by other structured exercise therapies.

Regarding safety, mild adverse events (CTCAE Grade 1)—including transient muscle soreness and increased knee or foot pain—were occasionally reported during the initial Tai Chi sessions, often attributable to improper posture [30,32]. These symptoms typically resolved after posture correction and appropriate technique instruction, with no further adverse events being observed. A comprehensive review of 24 Tai Chi RCTs involving 1794 participants found that Tai Chi did not incur a higher incidence of adverse events than either active or inactive control conditions, and in patients with heart failure, Tai Chi was associated with fewer adverse events compared to inactivity [42]. Thus, Tai Chi appears to be a safe and well-tolerated intervention relative to pharmacologic or invasive treatments. Nonetheless, incorrect execution may impose undue strain on joints and muscles; therefore, training under the guidance of a qualified instructor is essential to maximize therapeutic benefits and minimize injury risk.

The Yang style is the most widely practiced form of Tai Chi and is characterized by smooth, continuous movements, wide stances, deep knee flexion, and pronounced weight shifting [43]. However, the deep knee bending involved in Yang-style movements—particularly in lunge-like postures—may exacerbate knee pain, highlighting the importance of proper instruction by a qualified instructor [44]. In contrast, the Sun style features a narrower stance, less knee flexion, and light, agile movements using a “step up, step forward” approach. Compared to the Yang style, the Sun style is generally considered to place less mechanical stress on the knee joint, thereby reducing the likelihood of pain exacerbation [45]. Among the included studies, two trials [30,32] that reported increased knee pain during the initial Tai Chi sessions utilized the Yang style. This finding suggests the need for the careful consideration of participant characteristics, including the appropriate selection of Tai Chi style and proper instruction by qualified instructors to ensure safe and effective practice.

### 4.2. Limitations of the Review

This study has several limitations. First, in exercise-based interventions such as Tai Chi, the blinding of participants and instructors is inherently challenging, and the implementation of sham controls is difficult [46]. Consequently, the risk of measurement and reporting bias is elevated, potentially compromising the validity of the results. Future trials should address this by employing standardized RCT protocols and, where feasible, blinding outcome assessors and statisticians to minimize bias. Second, although 11 RCTs evaluating Tai Chi for knee pain were included, the overall methodological quality was generally low, warranting a cautious interpretation of the findings. Third, there was substantial heterogeneity in Tai Chi styles, program components, duration, and frequency across studies. This variability complicates data synthesis and highlights the need for standardized Tai Chi programs specifically tailored to knee pain management. Fourth, despite including 11 RCTs, only three studies were eligible for meta-analysis, precluding a reliable assessment of publication bias (e.g., funnel plots and Egger’s test). Established guidelines recommend publication bias assessment only when at least ten studies are included in a meta-analysis, as fewer studies substantially reduce reliability and interpretability [47]. Fifth, meta-analysis could not be conducted for three RCTs comparing Tai Chi with no intervention [29,31,33]—despite all using the WOMAC score—because of considerable heterogeneity in the reported data. Finally, 10 of the 11 included trials (90.1%) focused exclusively on knee osteoarthritis, limiting the generalizability of our findings to broader knee pain populations. Thus, it remains challenging to extrapolate these results to individuals with knee pain arising from other etiologies, such as trauma, post-exercise soreness, weight gain, or aging. Future high-quality RCTs should investigate Tai Chi’s effects on non-osteoarthritic knee pain, and an updated systematic review incorporating such studies would be highly valuable.

### 4.3. Implications for Future Studies

Exercise-based interventions, such as Tai Chi, present inherent challenges for participant and instructor blinding, which can compromise the methodological rigor of randomized controlled trials. To overcome these limitations, future research could adopt the following alternative designs: (1) Nationwide registry-based comparative analysis [48]: Utilize large-scale health databases to compare outcomes between individuals who practice Tai Chi and propensity-matched controls. This design offers extensive sample sizes and cost efficiency but depends on the accurate documentation of Tai Chi exposure; (2) Prospective cohort study [49]: Enroll a cohort of Tai Chi practitioners and a demographically similar non-practicing cohort, and follow both groups longitudinally to evaluate long-term effects. While this approach enables detailed subgroup analyses based on comprehensive health data, it is resource- and time-intensive and vulnerable to attrition bias; (3) Mixed-methods research [50]: Combine quantitative outcome measures with qualitative assessments of participant experiences, adherence, and acceptability. This integrative approach yields nuanced insights into factors that are difficult to capture quantitatively but requires specialized expertise in qualitative methodology and interpretation.

Advanced age is associated with declines in lower-limb strength and balance, which contribute to knee pain in older adults [51]. Tai Chi has been shown to significantly improve muscle strength and postural stability, thereby potentially reducing fall risk in this population [52]. However, practical constraints—such as limited time and space in clinical settings—may impede its widespread implementation. To maximize the therapeutic benefits of Tai Chi for knee pain among the elderly, future efforts should (1) develop and validate a standardized Tai Chi protocol specifically tailored to knee pain reduction; (2) establish a comprehensive instructor training program to ensure consistent delivery and safety of the intervention; and (3) conduct policy research to integrate Tai Chi offerings into public health center-level services, thereby enhancing accessibility in community settings.

## 5. Conclusions

Despite the heterogeneity in Tai Chi protocols and the generally low methodological quality of the included studies, this systematic review provides evidence that Tai Chi is effective in reducing knee pain and improving physical function when compared with inactive controls, such as health education or no intervention. However, in comparisons with active control interventions—including physical therapy and resistance training—no significant differences were observed in pain or functional outcomes, indicating that Tai Chi offers comparable benefits but is not superior to these established exercise-based therapies. From a clinical perspective, these findings suggest that Tai Chi is a safe, feasible, and low-impact exercise option that is particularly suitable for individuals who may have difficulty engaging in higher-intensity or weight-bearing activities. Its comparable effectiveness to conventional active interventions supports its use as an alternative or adjunctive therapy for managing knee pain, especially in older adults or those with functional limitations.

To more definitively establish the efficacy of Tai Chi for knee pain, future high-quality randomized controlled trials should adopt standardized intervention protocols and incorporate the blinding of outcome assessors and statisticians to reduce the risk of bias. Moreover, future studies should expand their focus to include individuals with knee pain stemming from non-osteoarthritic causes—such as trauma, post-exercise soreness, weight gain, or aging—to improve the generalizability of the findings.

## Figures and Tables

**Table 1:** Characteristics of the included studies.

First Author (Year)	Patient(s), Sample Size	Experimental Group (Intervention, Regimen)	Control Group (Intervention, Regimen)	Outcome Measurements	Main Result	Adverse Events
Song R (2003) [29], South Korea	Knee pain patients with knee osteoarthritis n = 43	Tai Chi n = 22; total of 36 sessions (three times a week for 12 weeks)	No intervention, n = 21	1. WOMAC score (1) Joint pain (2) Joint stiffness (3) Physical function 2. Function scale (1) Balance (2) Cardiovascular functioning 3. Muscle strength (1) Abdominal muscle strength (2) Knee muscle strength	1. (1) *p* < 0.05 (2) *p* < 0.05 (3) *p* < 0.01 2. (1) *p* < 0.01 (2) NS 3. (1) *p* < 0.01 (2) NS	n.r.
Brismée JM (2007) [30], United States	Knee pain patients with knee osteoarthritis n = 31	Tai Chi n = 18; total of 18 sessions (three times a week for 6 weeks)	Health education n = 13; total of 18 sessions (three times a week for 6 weeks)	1. VAS for knee pain 2. WOMAC score (1) Overall (2) Pain (3) Stiffness (4) Function 3. Function scale (knee ROM)	1. *p* < 0.05 2. (1) *p* < 0.05 (2) *p* < 0.05 (3) NS (4) *p* < 0.05 3. NS	(E) Minor muscle soreness and foot and knee pain after first session. No AEs were observed during the remaining sessions. (C) None.
Lee HJ (2008) [31], South Korea	Knee pain patients with knee osteoarthritis n = 41	Tai Chi n = 28; total of 16 sessions (twice a week for 8 weeks)	No intervention n = 13	1. WOMAC score (1) Total (2) Pain (3) Stiffness (4) Physical function 2. Functional scale (6 min walking test) 3. Quality of life (SF-36) (1) Total (2) Physical health (3) Mental health	1. (1) NS (2) *p* < 0.05 (3) NS (4) NS 2. *p* < 0.001 3. (1) *p* < 0.05 (2) *p* < 0.05 (3) *p* < 0.05	n.r.
Wang C (2009) [32], United States	Knee pain patients with knee osteoarthritis n = 40	Tai Chi n = 20; total of 24 sessions (twice a week for 12 weeks)	Stretching and health education n = 20; total of 24 sessions (twice a week for 12 weeks)	1. WOMAC score (1) Pain (2) Stiffness (3) Function 2. VAS for pain 3. Functional scale (1) 6 Minute Walk Test (2) Balance score (3) Chair stand time 4. Quality of life (SF-36) (1) Physical health (2) Mental health 5. Depressive scale (CES-D) 6. Self-Efficacy Score	1. (1) *p* < 0.001 (2) *p* < 0.05 (3) *p* < 0.01 2. *p* < 0.01 3. (1) NS (2) NS (3) *p* < 0.001 4. (1) *p* < 0.01 (2) NS 5. *p* < 0.01 6. *p* < 0.05	(E) One participant in the Tai Chi group reported an increase in knee pain at 2 weeks. This was resolved following the modification of that participant’s Tai Chi Technique. (C) None.
Wortley M (2013) [33], United States	Knee Pain patients with knee osteoarthritis n = 31	(E) Tai Chi n = 12; total of 20 sessions (two times a week for 10 weeks)	(C1) Resistance training n = 13; total of 20 sessions (two times a week for 10 weeks) (C2) No intervention n = 6	1. WOMAC score (1) Pain (2) Physical function (3) Stiffness 2. Functional scale (1) 6 min walking test (2) Time up and go test (3) Stair climb and descent test	1. (1) *p* < 0.01 in (C1), NS in (E) and (C2) (2) *p* < 0.05 in (C1), NS in (E) and (C2) (3) *p* < 0.05 in (E), *p* < 0.001 in (C1) and NS in (C2) 2. (1) NS in (E), (C1) and (C2) (2) *p* < 0.001 in (E), *p* < 0.01 in (C1) and NS in (C2) (3) NS in (E), (C1) and (C2)	n.r.
Tsai PF (2013) [34], United States	Knee pain patients with knee osteoarthritis n = 55	Tai Chi n = 28; total of 60 sessions (three times a week for 20 weeks)	Health education n = 27; 60 sessions (three times a week for 20 weeks)	1. WOMAC score (1) Pain score (2) Physical function (3) Stiffness 2. Function scale (sit to stand test) 3. MMSE score	1. (1) *p* < 0.01 (2) NS (3) *p* < 0.05 2. NS 3. NS	No AEs occurred in (E) and (C).
Chenchen W (2016) [35], United States	Knee pain patients with knee osteoarthritis n = 204	Tai Chi n = 106; total of 24 sessions (two times a week for 12 weeks)	Physical therapy n = 98; total of 24-48 sessions (2-4 sessions a week for 12 weeks)	1. WOMAC score (1) Pain (2) Physical function (3) Stiffness 2. Function scale (physical function) 3. Depression scale (BDI) 4. Medication use 5. Quality of life (SF-36)	1. (1) NS (2) NS (3) NS 2. NS 3. *p* < 0.01 4. NS 5. *p* < 0.05	No AEs occurred in (E) and (C).
Zhu Q (2016) [36], China	Knee pain patients with knee osteoarthritis n = 46	Tai Chi n = 23; total of 72 sessions (three times a week for 24 weeks)	Health education n = 23; total of 24 sessions (once a week for 24 weeks)	1. WOMAC score (1) Pain (2) Physical function (3) Stiffness 2. Function scale (SPPB)	1. (1) *p* < 0.01 (2) *p* < 0.01 (3) *p* < 0.001 2. *p* < 0.001	No AEs occurred in (E) and (C).
Buyukturan O (2019) [37], Turkey	Knee pain patients with partial anterior cruciate ligament injury n = 58	Tai Chi n = 29; total of 24 sessions (three times a week for 8 weeks)	No intervention n = 29	1. VAS for pain 2. Function scale (Lysholm Knee Scale) 3. Muscle strength (Biodex System 4-Pro) (1) Extension PT 60°/sec (2) Flexion PT 60°/sec (3) Extension PT 180°/sec (4) Flexion PT 180°/sec 4. Proprioception scale	1. *p* < 0.01 2. NS 3. (1) *p* < 0.05 (2) NS (3) *p* < 0.05 (4) NS 4. *p* < 0.001	n.r.
Liu J (2019) [38], China	Knee pain patients with knee osteoarthritis n = 52	Tai Chi n = 28; total of 60 sessions (five times a week for 12 weeks)	Health education n = 24; total of 60 sessions (five times a week for 12 weeks)	1. KOOS score (1) Pain (2) Symptoms (3) Daily living (4) Sport (5) Quality of life 2. Serum biomarker (1) BDNF (2) IFN-g (3) PD-1 (4) TIM-3 3. Functional connectivity changes in brain regions (1) PAG (2) VTA	1. (1) *p* < 0.01 (2) NS (3) NS (4) *p* < 0.01 (5) *p* < 0.01 2. (1) NS (2) *p* < 0.01 (3) *p* < 0.01 (4) NS (3) *p* < 0.01 3. (1) Tai Chi decreased right PAG rsFC with the medial orbital prefrontal cortex, and the decreased rsFC was associated with improvements in knee pain (2) There was also a significantly decreased rsFC between the left VTA and the medial orbital prefrontal cortex in the Tai Chi group	No AEs occurred in (E) and (C).
Hu X (2020) [39], China	Knee pain patients with knee osteoarthritis n = 92	Tai Chi n = 52; total of 72 sessions (three times a week for 24 weeks)	Health education n = 40; total of 24 sessions (once a week for 24 weeks)	1. VAS for pain 2. WOMAC score (1) Pain (2) Physical function (3) Stiffness 3. Proprioception scale (1) Plantarflexion of ankle (2) Dorsiflexion of ankle (3) Varus of ankle (4) Valgus of ankle (5) Flexion of knee (6) Extension of knee	1. NS 2. (1) NS (2) *p* < 0.05 (3) NS 3. (1) *p* < 0.05 (2) *p* < 0.05 (3) *p* < 0.05 (4) NS (5) *p* < 0.05 (6) NS	n.r.

AEs: adverse events; BDI: Beck Depression Inventory; (C): control intervention; CES-D: Depression Scale of the Center for Epidemiologic Studies; (E): experimental intervention; BDNF: brain-derived neurotrophic factor; IFN-g: interferon gamma; KOOS: Knee Injury and Osteoarthritis Outcome Score; MMSE: Mini-Mental State Examination; n.r.: not reported; NS: no significant differences between groups or before and after intervention; ROM: range of motion; PAG: periaqueductal gray; PD-1: programmed death-1; rsFC: resting-state functional connectivity; SF-36: Short-Form 36-Item Health Survey; SPPB: Short Physical Performance Battery; TIM-3: T-cell immunoglobulin and mucin-domain-containing molecule-3; VAS: Visual Analogue Scale; VTA: ventral tegmental area; WOMAC: Western Ontario and McMaster Universities Osteoarthritis Index.

**Table 2:** Details of Tai Chi program.

	Program Name	Details of Program
Song R (2003) [29]	12-form Tai Chi from Sun-style Tai Chi	20 min; 12-form Tai Chi from Sun-style Tai Chi (1) Warm-up exercise (2) Tai chi movement (3) Cool-down exercise
Brismée JM (2007) [30]	24-form Tai Chi from Yang-style Tai Chi	40 min; 24-form Tai Chi from Yang-style Tai Chi (1) Warm-up exercise for 5 min (2) Tai Chi movement for 30 min (3) Cool-down exercise for 5 min
Lee HJ (2008) [31]	18-form Tai Chi	60 min; 18-form Tai Chi (1) Warm-up exercise for 10 min (2) Tai Chi movement for 50 min - Raising the arms; - Opening the chest; - Painting a rainbow; - Separating the clouds; - Rolling the arms in a horse-riding stance; - Rowing the boat; - Carry ball in front of the shoulders; - Looking at the moon; - Pushing palms; - Cloud hands in a horse-riding stance; - Scooping the sea and searching the sky; - Pushing waves; - Flying dove spreads its wings; - Punching in horse stance; - Flying like wild geese; - Rotating wheel; - Stepping whilst bouncing a ball; - Balancing chi.
Wang C (2009) [32]	10-form Tai Chi from Yang-style Tai Chi	60 min; 10-form Tai Chi from Yang-style Tai Chi (1) Self-massage and a review of Tai Chi principles for 10 min (2) Tai Chi movement for 30 min (3) Breathing technique for 10 min (4) Relaxation for 10 min
Wortley M (2013) [33]	12-form Tai Chi from Yang-style Tai Chi	60 min; 12-form Tai Chi from Yang-style Tai Chi (1) The program began by learning the first two movements during the first session and then adding a new movement during each session for the first 5 weeks. (2) In each training session of the first few weeks, sufficient time was provided for practicing the new and previously learned movements. (3) During the last 5 weeks, participants also practiced the movements in the opposite direction to the original direction in order to similarly “load” both lower limbs.
Tsai PF (2013) [34]	12-form Tai Chi from Sun-style Tai Chi	Tai Chi started at 20 min per session and gradually increased to 40 min per session; 12-form Tai Chi from Sun-style Tai Chi (1) Stage 1 (30 sessions in the first 10 weeks) enabled them to learn the forms (2) Stage 2 (30 sessions in the second 10 weeks) enabled them to rehearse the forms
Chenchen W (2016) [35]	Developed classical Yang-style Tai Chi	60 min; developed classical Yang-style Tai Chi (1) Warm-up exercise (2) Review of Tai Chi principles (3) Tai Chi movement (4) Breathing techniques (5) Relaxation methods
Zhu Q (2016) [36]	8-form Tai Chi	60 min; 8-form Tai Chi (1) Withdraw and push (2) Fan through the back (3) Wave hands like clouds (4) Lift hand (5) Brush knee and twist steps (6) Step back to repulse monkey (7) Fair lady works at shuttles (8) Golden pheasant stands with one leg (right and left)
Buyukturan O (2019) [37]	10-form Tai Chi from Yang-style Tai Chi	60 min; 10-form Tai Chi from Yang-style Tai Chi (1) Warm-up exercise and a review of Tai Chi principles and techniques (2) Tai Chi movement (3) Breathing techniques (4) Various relaxation methods
Liu J (2019) [38]	24-form Tai Chi from Yang-Style Tai Chi	60 min; 24-form Tai Chi from Yang-style Tai Chi (1) Warm-up exercise for 10 min (2) Tai Chi movement for 30 min (3) Breathing techniques for 10 min (4) Relaxation for 10 min
Hu X (2020) [39]	Tai Chi	60 min; Tai Chi (1) Warm-up exercise for 5 min (2) Tai Chi movement for 50 min (3) Cool-down for 5 min

## Data Availability

The raw data supporting the conclusions of this article will be made available by the authors on request.

## Supplementary Materials

The following supporting information can be downloaded at https://www.mdpi.com/article/10.3390/healthcare13131615/s1. Table S1: PRISMA checklist; Table S2: Search strategies and search terms used in each database; Table S3: Details of active control interventions.
