# Supplementary material for: The Effectiveness and Safety of Tai Chi on Knee Pain: A Systematic Review and Meta-Analysis
Source: Healthcare (Basel). 2025 Jul 6;13(13):1615. doi: 10.3390/healthcare13131615 (PMC12249842; doi:10.3390/healthcare13131615)
Supplement: Supplementary file 1 [file healthcare-13-01615-s001.zip › Additional File 2. Search terms used in each database.pdf]

Supplement Table S2. Search terms used in each database and results

Medline via PubMed (15.3.2024.)

	Searches
#1	"Tai Ji"[MH] OR "Tai Chi"[TW] OR "Tai Chi Chuan"[TW] OR "Taiji"[TW] OR "Taijiquan"[TW] OR "T'ai Chi"[TW] OR "Tai Ji Quan"[TW]
#2	“knee pain”[MH]
#3	“randomized controlled trial”[PT] OR “controlled clinical trial”[PT] OR randomized[TIAB] OR placebo[TIAB] OR “drug therapy”[SH] OR randomly[TIAB] OR trial[TIAB] OR groups[TIAB]
#4	animals[MH] NOT humans[MH]
**#5**	**#1 AND #2 AND #3 AND #4 NOT #5**

Embase via Elsevier (15.3.2024.)

	Searches
#1	'Tai Ji’/exp OR ‘Tai Ji’:ab,ti OR ‘Tai Chi’/exp OR ‘Tai Chi’:ab,ti OR ‘Tai Chi Chuan’/exp OR ‘Tai Chi Chuan’:ab,ti OR ‘Taiji’/exp OR ‘Taiji’:ab,ti OR ‘Taijiquan’/exp OR ‘Taijiquan’:ab,ti OR ‘Tai Ji Quan’/exp OR ‘Tai Ji Quan’:ab,ti
#2	'knee pain’/exp OR ‘knee pain’:ab,ti
#3	'crossover procedure':de OR 'double-blind procedure':de OR 'randomized controlled trial':de OR 'single-blind procedure':de OR (random* OR factorial* OR crossover* OR cross NEXT/1 over* OR placebo* OR doubl* NEAR/1 blind* OR singl* NEAR/1 blind* OR assign* OR allocat* OR volunteer*):de,ab,ti
**#4**	**#1 AND #2 AND #3**

CENTRAL (15.3.2024.)

	Searches
#1	MeSH descriptor: [Tai Ji] explode all trees
#2	((Tai Chi) OR (Tai Chi Chuan) OR (Taiji) OR (Taijiquan) OR (Tai Ji Quan)):ti,ab,kw
#3	(knee pain):ab,ti
**#4**	**(#1 AND #2 AND #3) in Trials**

CINAHL (EBSCOhost) (15.3.2024.)

	Searches
#1	MH “Tai Chi”
#2	TX “Tai Ji" OR “Tai Chi Chuan” OR “Taiji” OR “Taijiquan” OR “Tai Ji Quan”
#3	#1 OR #2
#4	MH “Knee pain”
#5	MH “Clinical Trials”
**#6**	**#3 AND #4 AND #5**

Korean medical databases - ScienceON, Korean traditional knowledge portal, Korea Citation Index, Research Information Sharing Service, OASIS, and Korean Medical database (15.3.2024.)

	Searches
#1	“Tai Chi” OR “Tai Ji" OR “Tai Chi Chuan” OR “Taiji” OR “Taijiquan” OR “Tai Ji Quan” OR “태극권”

#2	“knee pain” OR “무릎 통증”
#3	“randomization” or “random” or “무작위”
#4	#1 AND #2 AND #3
