# Supplementary material for: The Effectiveness and Safety of Tai Chi on Knee Pain: A Systematic Review and Meta-Analysis
Source: Healthcare (Basel). 2025 Jul 6;13(13):1615. doi: 10.3390/healthcare13131615 (PMC12249842; doi:10.3390/healthcare13131615)
Supplement: Supplementary file 1 [file healthcare-13-01615-s001.zip › Additional File 3. Details of active control interventions.pdf]

Supplement Table S3. Details of active control interventions

	Active Control Interventions	Detalis of Program
Wang C [32] (2009)	Sretching exercise + Health education	60 min (20 min stretching exercises and 40 min health education), Wellness education and stretching program<br>(1) Stretching exercises involving the upper body, trunk and lower body, each stretch being held for 10 to 15 seconds<br>(2) Health education consist of ① Knee osteoarthritis as a disease; diet and nutrition; ③ Therapies to treat knee osteoarthritis; or ④ Physical and mental health education (e.g., recognizing and dealing with stress)
Wortley M [33] (2013)	Resistance training	60 min, Open-kinetic chain resistance training program<br>- The program included the following knee and hip exercises performed with ankle cuff weights: seated leg extension, standing hamstring curl, straight leg raise, standing hip abduction, standing hip adduction, standing hip flexion, standing calf raise<br>- Participants started with either a 5 lb or 10 lb ankle weight and progressed from two sets of eight repetitions to three sets of 12 repetitions during the first 6 weeks, and were allowed to increase the weight as needed during the final 4 weeks.
Chenchen W [35] (2016)	Physical therapy	30 min, Physical therapy protocol followed U.S. guidelines for knee osteoarthritis treatment<br>- At each session, the physical therapist examined the participant for adverse signs and symptoms before proceeding with manual therapy or exercise<br>- Patients were encouraged to also perform exercises at home. After 6 weeks, participants were instructed to continue exercises in 30-minute sessions 4 times per week for 6 weeks. These were monitored weekly by telephone by using standardized forms to ascertain frequency, exercises completed, adverse events, and adherence.
